# How Safe Is Safe for Marine Toxins Monitoring?

**DOI:** 10.3390/toxins8070208

**Published:** 2016-07-06

**Authors:** Luis M. Botana, Amparo Alfonso, Ines Rodríguez, Ana M. Botana, Maria del Carmen Louzao, Mercedes R. Vieytes

**Affiliations:** 1 Departamento de Farmacología, Facultad Veterinaria, Universidad de Santiago de Compostela, 27002 Lugo, Spain; amparo.alfonso@usc.es (A.A.); ines.rodriguez@usc.es (I.R.); mcarmen.louzao@usc.es (M.d.C.L.); 2 Facultad de Ciencias, Universidad de Santiago, 27002 Lugo, Spain; anamaria.botana@usc.es; 3 Departamento de Fisiología, Facultad Veterinaria, Universidad de Santiago de Compostela, 27002 Lugo, Spain; mmercedes.rodriguez@usc.es

**Keywords:** food safety, toxicity equivalency factor, mass spectrometry, monitoring, marine toxin

## Abstract

Current regulation for marine toxins requires a monitoring method based on mass spectrometric analysis. This method is pre-targeted, hence after searching for pre-assigned masses, it identifies those compounds that were pre-defined with available calibrants. Therefore, the scope for detecting novel toxins which are not included in the monitoring protocol are very limited. In addition to this, there is a poor comprehension of the toxicity of some marine toxin groups. Also, the validity of the current approach is questioned by the lack of sufficient calibrants, and by the insufficient coverage by current legislation of the toxins reported to be present in shellfish. As an example, tetrodotoxin, palytoxin analogs, or cyclic imines are mentioned as indicators of gaps in the system that require a solid comprehension to assure consumers are protected.

## 1. Monitoring of Toxins

The current monitoring method for marine toxins is based on an analysis with liquid chromatography separation coupled to a mass spectrometric detection [1] using interlaboratory validated methods [2].

The regulatory situation in Europe has been under critical scrutiny with regard to some contaminants such as endocrine disrupters [3], where toxicity was presumed in the absence of specific data demonstrating non toxicity. A critical letter was published simultaneously about this matter in several pharmacology and toxicology journals [4]. A similar situation is being criticized with regard to marine toxins, although with not such a relevant coordination by several journal editors. The work by the European Food Safety Authority (EFSA) working group has evidenced that even legally regulated, there is a lack of demonstrated toxic effect in humans by pectenotoxins [5] or yessotoxins [6]. On the other hand, there is a demonstrated presence of tetrodotoxin, a well known lethal compound [7], in shellfish [8]. Palytoxin and analogs, also very toxic to humans [9] were also reported to be present in shellfish [10]. Similarly, spirolides and cyclic imines, which were reported to be neurotoxic [11], orally absorbed and able to reach the central nervous system [12], are a frequently detected in shellfish [13]. With regard to marine toxins, it can be stated that some toxins are included in the legislation unless they are proven to be non toxic. This is the case as some of them are regulated while being non toxic (i.e., pectenotoxin), and others are not regulated even though they are very toxic (i.e., tetrodotoxin, palytoxin).

## 2. Analysis and Toxicity

The analytical control of the presence of marine toxins in shellfish [1,2] is based on a targeted method that only seeks to find predetermined compounds, while missing all other toxins that could be present in the sample [14]. Table 1 shows the toxins currently regulated and the suspected number of non regulated toxins. This new analytical monitoring approach for marine toxins has several important advantages, as it is possible to know the toxins profile and amount in a sample. Nevertheless, the pre-targeted monitoring has been a fundamental loss with regard to the capability to identify new toxins. When the mouse bioassay, that is regarded as a universal detector, was replaced by analytical methods [15], no new toxin can be detected unless new intoxications appear in consumers. A few years before the introduction of mass spectrometric analysis, the appearance of fast neurotoxins was reported in several parts of Canada and Europe [16]. Those fast neurotoxins were probably spirolides, and although their analysis was not required by the legislation, the bioassay was warning of something unusual present in the sample. This is no longer the case with the mass spectrometric detection.

The term SWATH, that stands for “sequential windowed acquisition of all theoretical fragment ion mass spectra” has been applied for the total search of compounds in a sample, eliminating the main bias of mass spectrometric analysis, the targeted nature of the analysis [21,22,23]. This is certainly an approach that can be adopted for marine toxins, although it will not be an easy task. There are several drawbacks that make this approach difficult, if possible at all. One is the complex nature of the matrix effect (clams, scallops, mussels, oysters, etc.) on the analysis [24], that prevent the development of universal protocol. In fact, even for a single toxin group it is difficult to use the same method in different matrices, and in many occasions a method is validated only for a limited number of matrices and toxins. Recent examples of interlaboratory exercices prove how difficult this matter can be, and a validated method [25] requires several refinements to extend it to more matrices [26] and toxins [27]. Also, mass spectrometric analysis for marine toxins face a significant challenge not only for matrices, but because marine toxins have a molecular range rather large, from small compounds such as domoic acid (MW 311 Da) to the largest non polimeric molecules in nature, such as palytoxin (MW 2680 Da) or maitotoxin (MW 3422 Da). In addition to the molecular weight range, the complexity of some groups is extreme; maitotoxin may have a theoretical number of 299 possible stereoisomers [17], and palytoxin “only” 264 stereogenic isomers [28]. Since each analog, even with a small stereogenic change, may be relevant from a toxicological standpoint, it is very difficult to decide what should be tested or not. The analysis of complex molecules, even with high resolution equipment, shows that different molecular charges and too many analogs for just one single toxin group, makes the analysis extremely complex. A recent study that combines high resolution mass spectrometric analysis of palytoxins combined with the high throughput capability of antibody-based flow cytometry [29] demonstrates that a routine analysis of certain toxin groups is close to impossible. The combination of limited antibody crossreactivity and the existance of so many analogs, such as palytoxins, ostreocins or ovatoxins create a situation of many potential toxic molecules and no discrimination capability, with very limited output in terms of toxicity. Although this problem can be avoided with functional assays [30], todays legislation do not contemplate such an option.

Another aspect to consider in non targeted mass spectrometric analysis is the influence of uncontrolled factors in the quality of the study, specially if the method is to be translated to equivalent results in another laboratory through a validated method [31]. Since the control of toxins has to be quantitative, the monitoring analysis must be able to detect toxins levels, below the legal threshold, that permit to release a product to the market. An analysis in multiple reaction monitoring (MRM) mode with a triple quadrupole, which would allow only targeted screening, could hardly be replaced by a scan analysis, that adds to the system the low sensitivity of a quantitation in this mode and requires an extremely efficient separation of the molecules by chromatography. Generally, LC-MRM is used for strictly targeted screening, and untargeted screening can be done with high resolution mass spectrometers (HRMS). Nevertheles, there is a limitation due to the lower speed and sensitivity of a HRMS when compared to triple quadrupoles [32] although the most modern (and expensive) HRMS have a high sensitivity, that equals that of triple quadruples [33]. Also the fact that a high resolution ion spectrum requires to predefine the criteria to select mass precursors for the trigger events, creates a contradiction between exclusion times (to reduce peak numbers) and maximum peak quantitation (the more trigger events the longer cycle times). Therefore, a SWATH approach would requires a narrow mass range for the equipment to be able to cope with many compounds [21], and this is not compatible with the mass range required for marine toxins. Moreover, a SWATH approach would requires a very extensive database, which might be is partially available for some of the toxin groups (azaspiracids [34], yessotoxins [35], dinophysistoxins, tetrodotoxins), but not for others (ostreocins, ovatoxins [36], palytoxins, ciguatoxins [37]).

**Figure 1:**
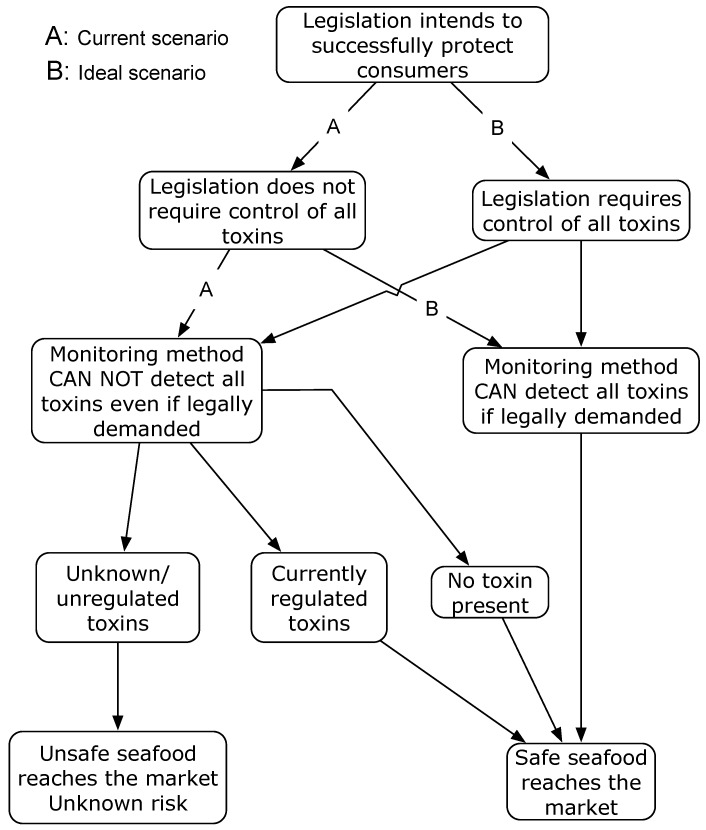
Uncertainty scheme of marine toxins analytical monitoring under the current situation and under an ideal situation.

In the best of the situations, where a very fast high resolution mass spectrometer with very low detection limit can analyze several hundred compounds simultaneously, there would be is still a fundamental limitation based on the lack of standards to identify each individual mass. Even if these standards were available (we are far from that), there would not be certified standards for a proper calibration, not to mention the need of an interlaboratory quantification exercise that validates the method. So, to be realistic, this will not happen in quite some time. The current situation is reflected and summarized in Figure 1.

## 3. Toxicity Itself

Finally, for each compound identified, there would be it is needed a toxicity equivalency factor (TEF) that converts the calculated value to a reference compound of the same toxin group, as toxicity values are referred as equivalents of a reference compound [1]. The problem of the estimation of TEF is another challenge that requires a combined effort of toxicologists and analytical chemists, and it is not resolved for most the toxins, since the toxicology is not understood, even for toxin groups that are very common. An example of this complexity is the worldwide common group of diarrheic phosphatase inhibitors, such as dinophysistoxins (DTX) and okadaic acid (OA) [38]. These toxins were considered to be diarrheic as a consequence of a mechanism that would modify the integrity of the gap junctions in the intestinal epithelium, hence increasing paracellular permeability [39]. But there are several phosphatase inhibitors that do not cause diarrhea, but hepatotoxicity, such as tautomycin, [40] microcystins, calyculin A or nodularins [41]. It has been reported that severe diarrhea induced by OA and DTX is not associated with mucosal damage [42]. Also, a non-phosphatase inhibitor, methyl okadaate, has a higher potency than OA to disrupt F-actin [43], and in general there is no study that links phosphatase inhibition with tight junction integrity and diarrhea [44]. On the other hand, it has been proposed that OA-induced diarrhea might be associated to modes of action related to neurotransmitters [45].

Given the poor toxicological comprehension of some toxin groups, it is therefore difficult to define a TEF. The mechanism of action of azaspiracids has not been elucidated yet, although several potential candidates were outlined [46], and this makes difficult to propose a TEF for this group. Also, it is unclear how relevant it is the oral toxicity of a toxin group in mice compared to the potency of the same group in human receptors in vitro, as it is the case for saxitoxin and analogs [47,48]. It seems to be the case though that most of the very toxic compounds, such as palytoxin, are far less toxic by the oral route [18], but they are still quite toxic. Another example is the cardiotoxicity associated to some toxins, such as yessotoxin, azaspiracids or domoic acid [49,50,51,52]. The majority of these results are recent and were not included in the risk assessment performed at the time by EFSA [19].

The rapid alert system in the EU (RASFF, Food and Feed Safety Alerts [53]) shows for marine toxins the same frequency of incidences before and after the introduction of LC-MS in 2011 (i.e., 14 in 2010, 14 in 2014, 8 in June 2016), and two serious notifications for ciguatoxin, in 2012 (Germany) and 2015 (France) [54,55]. RASFF shows no result on tetrodotoxin, although a serious intoxication did occur [56].

Therefore, the conclusion is that the combination of unclear TEFs, lack of sufficient standards, a targeted analytical method for monitoring and a poorly defined legislation that does not include several relevant toxin groups, makes the consumer situation for marine toxicity safety, uncertain.

## Figures and Tables

**Table 1:** Monitored toxins vs. toxins not required to be monitored [1,17,18,19].

Toxin Group	Proven to Be Toxic to Humans?	Number of Analogs Monitored [1]	Number of Analogs not Monitored #
Okadaic acid	YES	4 (DTX3 is a group of molecules)	0
Azaspiracid	YES	3	40
Domoic acid	YES	2	0 (traces of others)
Saxitoxin	YES	14	30
Bevetoxin	YES	Non polar (Mouse bioassay)	Polar compounds are undetected due to poor extraction
Ciguatoxin	YES	0 (two in the US)	15
Tetrodotoxin	YES	0	15
Palytoxin	YES	0	Unkown. In theory many billions [17].
Cyclic imines	NO *	0	40
Yessotoxin	NO **	4	50
Pectenotoxin	NO	2	15

# Numbers are approximate. There are many analogs reported in some toxin groups, but the toxicity and the abundancy of each analog is either low or unknown. * No evidence reported in humans, mechanistically they might be toxic in the long term [11,12]. ** Reported cardiotoxicity in animals [20], toxic episode never reported in humans.

## References

[1] EU (2011). Commission regulation (EU) No 15/2011 of 10 January 2011 amending Regulation (EC) No 2074/2005 as regards recognised testing methods for detecting marine biotoxins in live bivalve molluscs. Offi. J. Eur. Communities.

[2] These A., Klemm C., Nausch I., Uhlig S. (2011). Results of a European interlaboratory method validation study for the quantitative determination of lipophilic marine biotoxins in raw and cooked shellfish based on high-performance liquid chromatography-tandem mass spectrometry. Part I: Collaborative study. Anal. Bioanal. Chem..

[3] Dietrich D.R., von Aulock S., Marquardt H., Blaauboer B., Dekant W., Kehrer J., Hengstler J., Collier A., Gori G.B., Pelkonen O. (2013). Scientifically unfounded precaution drives European Commission's recommendations on EDC regulation, while defying common sense, well-established science and risk assessment principles. Food Chem. Toxicol..

[4] Dekant W., Kehrer J.P. (2013). Scientifically unfounded precaution drives European Commission's recommendations on EDC regulation, while defying common sense, well-established science and risk assessment principles. Toxicol. Lett..

[5] EFSA (2009). Scientific Opinion on marine biotoxins in shellfish- Pectenotoxin group. EFSA Panel on Contaminants in the Food Chain (CONTAM). EFSA J..

[6] EFSA (2008). Scientific Opinion on marine biotoxins in shellfish- Yessotoxin group. EFSA Panel on Contaminants in the Food Chain (CONTAM). EFSA J..

[7] Russell F.E. (1975). Poisonous and venomous marine animals and their toxins. Ann. N. Y. Acad. Sci..

[8] Vlamis A., Katikou P., Rodriguez I., Rey V., Alfonso A., Papazachariou A., Zacharaki T., Botana A.M., Botana L.M. (2015). First Detection of Tetrodotoxin in Greek Shellfish by UPLC-MS/MS Potentially Linked to the Presence of the Dinoflagellate Prorocentrum minimum. Toxins (Basel).

[9] Katikou P., Vlamis A. (2014). Palytoxin and Analogs: Ecobiology and Origin, Chemistry, and Chemical Analysis.

[10] Aligizaki K., Katikou P., Nikolaidis G., Panou A. (2008). First episode of shellfish contamination by palytoxin-like compounds from Ostreopsis species (Aegean Sea, Greece). Toxicon.

[11] Molgo J., Girard E., Benoit E., Botana L.M. (2007). The cyclic imines: An insight into this emerging group of bioactive marine toxins. Phytotoxins, Chemistry and Biochemistry.

[12] Otero P., Alfonso A., Rodriguez P., Rubiolo J.A., Cifuentes J.M., Bermudez R., Vieytes M.R., Botana L.M. (2012). Pharmacokinetic and toxicological data of spirolides after oral and intraperitoneal administration. Food Chem. Toxicol..

[13] Villar Gonzalez A., Rodriguez-Velasco M.L., Ben-Gigirey B., Botana L.M. (2006). First evidence of spirolides in Spanish shellfish. Toxicon.

[14] Botana L.M. (2012). A perspective on the toxicology of marine toxins. Chem. Res. Toxicol..

[15] Botana L.M., Botana L.M. (2008). The mouse bioassay as a universal detector. Seafood and Freshwater Toxins: Pharmacology, Physiology and Detection.

[16] Cembella A.D., Quilliam M.A., Lewis N.I., Bauder A.G., Wright J.L.C. Identifying the Planktonic Origin and Distribution of Spirolides in Coastal Nova Scotian Waters. http://epic.awi.de/11292/.

[17] Nicolaou K.C., Frederick M.O., Aversa R.J. (2008). The continuing saga of the marine polyether biotoxins. Angew. Chem..

[18] Munday R., Botana L.M. (2014). Toxicology of seafood toxins: A critical review. Seafood and Freshwater Toxins: Pharmacology, Physiology and Detection.

[19] EFSA (2009). Marine biotoxins in shellfish-Summary on regulated marine biotoxins. EFSA J..

[20] Ferreiro S.F., Carrera C., Vilarino N., Louzao M.C., Santamarina G., Cantalapiedra A.G., Botana L.M. (2015). Acute cardiotoxicity evaluation of the marine biotoxins OA, DTX-1 and YTX. Toxins (Basel).

[21] Scheidweiler K.B., Jarvis M.J., Huestis M.A. (2015). Nontargeted SWATH acquisition for identifying 47 synthetic cannabinoid metabolites in human urine by liquid chromatography-high-resolution tandem mass spectrometry. Anal. Bioanal. Chem..

[22] Arnhard K., Gottschall A., Pitterl F., Oberacher H. (2015). Applying “Sequential Windowed Acquisition of All Theoretical Fragment Ion Mass Spectra” (SWATH) for systematic toxicological analysis with liquid chromatography-high-resolution tandem mass spectrometry. Anal. Bioanal. Chem..

[23] Roemmelt A.T., Steuer A.E., Poetzsch M., Kraemer T. (2014). Liquid chromatography, in combination with a quadrupole time-of-flight instrument (LC QTOF), with sequential window acquisition of all theoretical fragment-ion spectra (SWATH) acquisition: Systematic studies on its use for screenings in clinical and forensic toxicology and comparison with information-dependent acquisition (IDA). Anal. Chem..

[24] Ciminiello P., Dell’Aversano C., Dello Iacovo E., Fattorusso E., Forino M., Tartaglione L., Rossi R., Soprano V., Capozzo D., Serpe L. (2011). Palytoxin in seafood by liquid chromatography tandem mass spectrometry: Investigation of extraction efficiency and matrix effect. Anal. Bioanal. Chem..

[25] Lawrence J.F., Niedzwiadek B., Menard C. (2004). Quantitative determination of paralytic shellfish poisoning toxins in shellfish using prechromatographic oxidation and liquid chromatography with fluorescence detection: Interlaboratory study. J. AOAC Int..

[26] Turner A.D., Hatfield R.G. (2012). Refinement of AOAC Official Method 2005.06 liquid chromatography-fluorescence detection method to improve performance characteristics for the determination of paralytic shellfish toxins in king and queen scallops. J. AOAC Int..

[27] Ben-Gigirey B., Rodriguez-Velasco M.L., Gago-Martinez A. (2012). Extension of the validation of AOAC Official Method 2005.06 for dc-GTX2,3: Interlaboratory study. J. AOAC Int..

[28] Katikou P., Botana L.M. (2007). The chemistry of palytoxins and ostreocins. Phytotoxins, Chemistry and Biochemistry.

[29] Fraga M., Vilarino N., Louzao M.C., Fernandez D.A., Poli M., Botana L.M. (2016). Detection of palytoxin-like compounds by a flow cytometry-based immunoassay supported by functional and analytical methods. Anal. Chim. Acta.

[30] Botana L.M., Alfonso A., Botana A., Vieytes M.R., Vale C., Vilariño N., Louzao M.C. (2009). Functional assays for marine toxins as an alternative, high-throughput screening solution to animal tests. Trends Anal. Chem..

[31] Otero P., Alfonso A., Alfonso C., Rodriguez P., Vieytes M.R., Botana L.M. (2011). Effect of uncontrolled factors in a validated liquid chromatography-tandem mass spectrometry method question its use as a reference method for marine toxins: Major causes for concern. Anal. Chem..

[32] Krock B., Tillmann U., John U., Cembella A. (2008). LC-MS-MS aboard ship: Tandem mass spectrometry in the search for phycotoxins and novel toxigenic plankton from the North Sea. Anal. Bioanal. Chem..

[33] Zendong Z., McCarron P., Herrenknecht C., Sibat M., Amzil Z., Cole R.B., Hess P. (2015). High resolution mass spectrometry for quantitative analysis and untargeted screening of algal toxins in mussels and passive samplers. J. Chromatogr. A.

[34] Hess P., McCarron P., Krock B., Kilcoyne J., Miles C.O., Botana L.M. (2014). Azaspiracids: Chemistry, biosynthesis, metabolism, and detection. Seafood and Freshwater Toxins: Pharmacology, Physiology and Detection.

[35] Ciminiello P., Dell’Aversano C., Fattorusso E., Forino M., Magno S., Poletti R. (2002). The detection and identification of 42,43,44,45,46,47,55-heptanor-41-oxoye ssotoxin, a new marine toxin from Adriatic shellfish, by liquid chromatography-mass spectrometry. Chem. Res. Toxicol..

[36] Tartaglione L., Mazzeo A., Dell’Aversano C., Forino M., Giussani V., Capellacci S., Penna A., Asnaghi V., Faimali M., Chiantore M. (2016). Chemical, molecular, and eco-toxicological investigation of Ostreopsis sp. from Cyprus Island: Structural insights into four new ovatoxins by LC-HRMS/MS. Anal. Bioanal. Chem..

[37] Lewis R.J. (2006). Ciguatera: Australian perspectives on a global problem. Toxicon.

[38] Yasumoto T., Oshima Y., Yamaguchi M. (1978). Occurrence of a new type of toxic shellfish in Japan and chemical properties of the toxin. Bull. Jpn. Soc. Sci. Fish..

[39] Tripuraneni J., Koutsouris A., Pestic L., De Lanerolle P., Hecht G. (1997). The toxin of diarrheic shellfish poisoning, okadaic acid, increases intestinal epithelial paracellular permeability. Gastroenterology.

[40] MacKintosh C., Klumpp S. (1990). Tautomycin fro the bacterium Streptomyces verticillatus, another potent and specific inhibitor of protein phosphatases 1 and 2A. FEBS Lett..

[41] Craig M., Holmes C.F.B., Botana L.M. (2000). Freshwater hepatotoxins. Microcystin and Nodularin, mechanisms of toxicity and effects on health. Seafood and Freshwater Toxins: Pharmacology, Physiology and Detection.

[42] Vieira A.C., Rubiolo J.A., Lopez-Alonso H., Cifuentes J.M., Alfonso A., Bermudez R., Otero P., Vieytes M.R., Vega F.V., Botana L.M. (2013). Oral toxicity of okadaic acid in mice: Study of lethality, organ damage, distribution and effects on detoxifying gene expression. Toxins (Basel).

[43] Espina B., Louzao M.C., Cagide E., Alfonso A., Vieytes M.R., Yasumoto T., Botana L.M. (2010). The methyl ester of okadaic acid is more potent than okadaic acid in disrupting the actin cytoskeleton and metabolism of primary cultured hepatocytes. Br. J. Pharmacol..

[44] Munday R. (2013). Is protein phosphatase inhibition responsible for the toxic effects of okadaic Acid in animals?. Toxins (Basel).

[45] Louzao M.C., Fernandez D.A., Abal P., Fraga M., Vilarino N., Vieytes M.R., Botana L.M. (2015). Diarrhetic effect of okadaic acid could be related with its neuronal action: Changes in neuropeptide Y. Toxicol. Lett..

[46] Twiner M., Hess P., Doucette G.J., Botana L.M. (2014). Azaspiracids: Toxicology, pharmacology and risk assessment. Seafood and Freshwater Toxins: Pharmacology, Physiology and Detection.

[47] Munday R., Thomas K., Gibbs R., Murphy C., Quilliam M.A. (2013). Acute toxicities of saxitoxin, neosaxitoxin, decarbamoyl saxitoxin and gonyautoxins 1&4 and 2&3 to mice by various routes of administration. Toxicon.

[48] Alonso E., Alfonso A., Vieytes M.R., Botana L.M. (2016). Evaluation of toxicity equivalent factors of paralytic shellfish poisoning toxins in seven human sodium channels types by an automated high throughput electrophysiology system. Arch. Toxicol..

[49] Vieira A.C., Cifuentes J.M., Bermudez R., Ferreiro S.F., Castro A.R., Botana L.M. (2016). Heart Alterations after Domoic Acid Administration in Rats. Toxins (Basel).

[50] Ferreiro S.F., Vilarino N., Carrera C., Louzao M.C., Cantalapiedra A.G., Santamarina G., Cifuentes J.M., Vieira A.C., Botana L.M. (2016). Subacute Cardiovascular Toxicity of the Marine Phycotoxin Azaspiracid-1 in Rats. Toxicol. Sci..

[51] Ferreiro S.F., Vilarino N., Carrera C., Louzao M.C., Cantalapiedra A.G., Santamarina G., Cifuentes J.M., Vieira A.C., Botana L.M. (2016). Subacute Cardiotoxicity of Yessotoxin: In vitro and in vivo Studies. Chem. Res. Toxicol..

[52] Vranyac-Tramoundanas A., Harrison J.C., Sawant P.M., Kerr D.S., Sammut I.A. (2011). Ischemic cardiomyopathy following seizure induction by domoic Acid. Am. J. Pathol..

[53] RASFF. https://webgate.ec.europa.eu/rasff-window/portal/.

[54] Otero P., Perez S., Alfonso A., Vale C., Rodriguez P., Gouveia N.N., Gouveia N., Delgado J., Vale P., Hirama M. (2010). First toxin profile of ciguateric fish in Madeira Arquipelago (Europe). Anal. Chem..

[55] Mattei C., Vetter I., Eisenblatter A., Krock B., Ebbecke M., Desel H., Zimmermann K. (2014). Ciguatera fish poisoning: A first epidemic in Germany highlights an increasing risk for European countries. Toxicon.

[56] Rodriguez P., Alfonso A., Vale C., Alfonso C., Vale P., Tellez A., Botana L.M. (2008). First toxicity report of tetrodotoxin and 5,6,11-trideoxyTTX in the trumpet shell Charonia lampas lampas in Europe. Anal. Chem..

